# Effect of Deep Slow Breathing on Pain-Related Variables in Osteoarthritis

**DOI:** 10.1155/2019/5487050

**Published:** 2019-06-03

**Authors:** Kalee L. Larsen, Lorrie R. Brilla, Wren L. McLaughlin, Ying Li

**Affiliations:** Health and Human Development Department, Western Washington University, Bellingham, WA 98225, USA

## Abstract

This study evaluated the effect of a six-week deep slow breathing (DSB) program on pain, physical function, and heart rate variability (HRV) in subjects with lower extremity joint pain. Twenty subjects were assigned into training (*n* = 10) and control (*n* = 10) groups. The training group participated in a six-week DSB program consisting of weekly training sessions and at-home breathing exercises. DSB exercises focused on prolonging the exhalation and the pause following exhalation. The Western Ontario and McMaster Universities Osteoarthritis Index (WOMAC) was used to assess pain and physical function, and HRV data were obtained before and after intervention. Results revealed no significant interactions between group and time for any of the variables. There was no significant main effect for group, but there was a significant main effect (*p* < 0.025) and a large effect size for time on both pain (*η*_*p*_^2^ = 0.454) and physical function (*η*_*p*_^2^ = 0.506). There were no significant main effects (*p* > 0.017) for group and time on LF power (group *η*_*p*_^2^ = 0.039, time *η*_*p*_^2^ = 0.061), HF power (group *η*_*p*_^2^ = 0.039, time *η*_*p*_^2^ = 0.039), and LF/HF ratio (group *η*_*p*_^2^ = 0.036, time *η*_*p*_^2^ = 0.169). Results indicated that the six-week DSB program was not sufficient to alleviate pain or improve physical function in subjects with lower extremity joint pain. Although the pain was not alleviated, other beneficial effects such as better coping with the pain were reported in the majority of training subjects. As this is the first study to examine the use of DSB for lower extremity joint pain and dysfunction, further research is needed to investigate the efficacy and applicability of DSB.

## 1. Introduction

Arthritis is the leading cause of disability among adults in the United States, affecting an estimated 50 percent of people aged 65 and older. The disease is associated with other health disorders such as diabetes, hypertension, obesity, and cancer. Half of those with arthritis also suffer from cardiovascular disease [1], the leading cause of death among both men and women in the United States [2]. Osteoarthritis (OA) is the most common form of arthritis, mainly affecting the hands, hips, and knees. Those suffering from OA experience a great deal of pain, limiting physical activity, and decreasing quality of life. Fear of pain or worsening symptoms may discourage those with OA from beneficial exercise, leading to other health problems associated with OA such as weight gain and heart disease [1]. Intervention goals for OA include reducing pain, improving quality of life, losing excess weight, and making lifestyle changes that improve health such as diet and physical activity. Because pain is the major limiting factor for those with OA, most interventions are aimed at reducing pain. However, surgical interventions are expensive and nonsurgical interventions, such as analgesics and physical therapy, have low efficacy [3]. Some interventions, such as pain medications, may produce other health complications including damage to the liver, kidneys, and gastrointestinal tract [4, 5].

There is a need for effective nonsurgical interventions. Exercise has been proposed as an effective and beneficial intervention for those with OA. Exercise reduces pain and improves function, quality of life, mood, and confidence to manage health [1]. Despite the benefits of exercise, 60 percent of people suffering with the disease do not adhere to physical activity guidelines and 23 percent are categorized as physically inactive. This decrease in physical activity in those with OA may be a result of pain or fear of worsening symptoms with exercise [6]. Reduced physical activity progresses OA by increasing stiffness and weakness in the joints and eliciting metabolic acidosis and chronic inflammation, making exercise even more challenging [5]. Aerobics and resistance training are effective nonsurgical interventions but many with OA will not participate, and therefore, alternative exercises must be evaluated. Breathing exercises have been used for years in a clinical setting to reduce pain and improve health, especially in labor and delivery [7]. However, they have not been studied as an intervention for OA.

Breathing plays an important role in pain signalling and autonomic nervous system (ANS) activation, emotion regulation, acid/base balance, and anti-inflammatory processes [8–12]. Recent studies suggest that deep slow breathing (DSB) relieves pain [9] and sleep disruption from pain [12] and improves mood [8]. Studies in healthy subjects reveal that DSB reduces pain by increasing the pain threshold, increasing parasympathetic (P-ANS) activity [9], decreasing (S-ANS) sympathetic activity [8], and altering pCO_2_ and pH [13]. Breathing also improves mood, which would largely impact those with OA, since many suffer from depression as well [8]. Deep slow breathing has not been studied as an intervention for OA but may be an effective method for improving pain, function, and mood. This may be appealing to patients with OA because the time commitment and cost are less than those in traditional therapeutic measures. In addition, DSB puts no mechanical stress on the joints and may be relaxing for those with OA. As symptoms improve with the use of DSB, those with OA may be more likely to participate in other physical activities, resulting in further health benefits.

The purpose of our study was to determine if a significant difference existed in joint pain perception and autonomic activity following a six-week breathing exercise program. We hypothesized that the DSB program would lead to significant differences in pain, physical function, and autonomic activity between training and control groups [14]. As the prevalence of OA rises, effective interventions that target pain are highly valuable. The role of DSB in improving chronic pain is not well researched. Specifically, the effect of DSB has not been studied in patients with joint pain. A better understanding of the effects and mechanisms of DSB may provide novel and practical approaches to treating joint pain.

## 2. Materials and Methods

### 2.1. Description of Study Population

Twenty subjects participated in the study. Subjects had been diagnosed by their physician with knee osteoarthritis (OA) and had received a normative score of less than 50 on the American Academy of Orthopaedic Surgeons (AAOS) Hip and Knee Questionnaire. Subjects were recruited from local senior centers and university campus through informational posters following approval of the study from the University Human Subjects' Committee. Subjects with unilateral joint pain had no history of joint replacement surgery, and subjects with bilateral joint pain that had undergone joint replacement surgery on only one joint were allowed to participate in the study. The joint that had not been operated on was used for assessment with the Western Ontario and McMaster Universities Osteoarthritis Index (WOMAC). For subjects with bilateral joint pain who had not undergone joint replacement surgery, the extremity with more severe pain, determined by the WOMAC pain score, was used for WOMAC assessment. No subjects had undergone joint surgery within the last six months. All subjects obtained medical clearance from a physician to participate in the study. Breathing practices were novel to the subjects.

### 2.2. Design of the Study

This study utilized a pretest-posttest experimental design in which subjects were assigned to either the control or training group based on their availability for training. Those who could meet the time commitment for the training group were assigned to the training group on a first come-first serve basis.

### 2.3. Experimental Protocol

#### 2.3.1. Training Protocol

The study was conducted over a six-week period. All subjects were instructed not to alter their diet or physical activity level during the study. Three-day diet and three-day physical activity logs were completed before and after breathing intervention. These logs were used to verify that no substantial changes were made in diet or physical activity. A medication log was submitted before and after the study. Subjects were instructed to notify the instructor if they began a new pain medication during the six-week study period. The control group did not attend weekly breathing training sessions.

#### 2.3.2. Weekly Breathing Training

Subjects were instructed to wear comfortable and loose-fitting clothes for 30-minute breathing sessions. Subjects were divided into groups of 2–4 subjects for each session. During DSB training, all subjects sat upright on a chair with back support. Subjects were informed of the discomfort or “air hunger” they may experience during DSB training. Subjects were assured that they could take a break from the breathing exercises if they felt dizzy or out of breath. All training sessions were conducted at the local senior center.

During the first twenty minutes of breathing training, a script or outline was used to guide subjects through the breathing exercises. A script was used during the first week and outlines were used for the remaining weeks so that the trainer could more easily monitor the subjects. The script and outlines were timed, and all subjects received the same training. The focus of the breathing exercises was on inhaling deeply, prolonging the exhalation, and performing the expiratory pause. Each week, subjects were given a focus topic to keep their interest in the program. Weeks one and two focused on awareness of breathing, weeks three and four focused on relaxation and tension release, and weeks five and six focused on breath control. The control group was not given any education or training.

Specific parameters for controlling depth and frequency of breathing were not used in this study. The goal of the breathing exercises was to increase the depth and decrease the frequency of respiration from initial values for each subject. The expiratory pause aided subjects in reducing breath frequency. The remaining ten minutes of training was used to record respiratory rate and expiratory pause for each subject. Subjects performed the expiratory pause following normal inhalation and exhalation. Following exhalation, subjects plugged their nose with their fingers and closed their mouth. This was held until the subject felt the very first urge to inhale. The amount of time that a subject could hold this pause was recorded as the expiratory pause [13].

#### 2.3.3. At-Home Breathing Protocol

DSB subjects were instructed to complete the DSB exercises with the expiratory pause at home five days a week, 20–30 minutes a day [13]. DSB subjects were given written instructions and a link to an instructional YouTube video on the expiratory pause to help them practice during the week. DSB subjects were educated on the importance of relaxation during DSB and instructed not to complete the breathing exercises while doing an attentive task such as reading, watching TV, conversing with family, or other tasks. Subjects were instructed to do the breathing exercises in a quiet room in an upright, seated position on a chair with back support. Both feet were to be placed on the floor. On the day of the weekly group training session, subjects were not required to complete additional DSB at home. Subjects kept a training or practice log and returned the log to the trainer each week to ensure compliance with the study.

### 2.4. Measurement Techniques and Procedures

#### 2.4.1. Western Ontario and McMaster Universities Osteoarthritis Index

In order to evaluate the effect of DSB on pain and physical function in those with OA, a valid and reliable measurement must be used. The WOMAC is one of the most commonly used self-reported measures of lower extremity symptoms and function. It was specifically designed to evaluate pain, function, and stiffness in subjects with OA of the hip and knee. The WOMAC is supported as a valid and reliable measurement of pain and physical function in subjects with hip or knee OA [15–19]. This measurement tool may be applied to studies examining the efficacy of pain interventions for hip and knee OA.

In this study, permission to use the WOMAC visual analog scale (VAS) was granted by Dr. Bellamy. The WOMAC VAS [20] was used to measure pain (five items) and physical function (17 items). Responses were based on the 1–100 mm VAS. Responses were scored using a ruler to measure the distance in millimeters from the left end to the subject's pencil mark. Scores for each item were summed to obtain scores for each category: pain and physical function. Higher scores indicated worse pain and physical function. The minimal clinically important improvement (MCII) values, defined as the smallest change in measurement that indicates substantial improvement in symptoms for hip and knee OA, were defined as −15.3 and −19.9 mm, respectively, for pain and −7.9 and −0.1 mm, respectively, for physical function [21].

#### 2.4.2. Heart Rate Variability

Heart rate variability (HRV) may be used to evaluate the efficacy of DSB in those with OA. HRV is recognized as an indicator of ANS activity and may be used to assess the balance between P-ANS and S-ANS activity [22]. Since previous studies have shown a link between breathing, changes in sympathovagal balance, and decreased pain [8, 9], HRV was a good measurement tool for this study.

HRV was measured using Biopac Systems MP150 [23]. HRV measurements were taken with a standard 3-electrode, 1-lead EKG setup which examined lead II. AcqKnowledge software [24] was used for data recording, collection, and data reduction. HRV variables were obtained from the raw EKG data, which used algorithms following the frequency domain guidelines established by the Task Force of the European Society of Cardiology and The North American Society of Pacing and Electrophysiology. Power frequency for HRV variables were defined by Task Force guidelines as very low frequency (VLF), ≤0.04 Hz, low frequency (LF), 0.04–0.15 Hz, high frequency (HF), 0.15–0.4 Hz, and very high frequency (VHF), ≥0.4 Hz [22]. LF power indicates sympathetic ANS activity, and HF power indicates parasympathetic ANS activity [9].

For HRV assessment, within 48 hours of completing the 6-week training program, subjects were instructed to lie still on an examination table in the laboratory for five minutes. The laboratory was kept quiet and at a comfortable temperature. Three electrodes were configured to examine lead II. The AcqKnowledge software [24] recorded HRV data for five minutes. The subject was instructed to close their eyes and remain still and relaxed during the measurement. The data collection was terminated and restarted if the subject talked or moved during collection.

#### 2.4.3. Expiratory Pause

The expiratory pause was measured as an indicator of breathing training efficacy. The expiratory pause was measured using a SportLine 220 stopwatch. Subjects were taught to perform the expiratory pause by closing their eyes and inhaling and exhaling as usual. Following exhalation, subjects were instructed to close their mouth, plug their nose, and hold this until they felt the very first urge to breathe. At the very first urge to breathe, subjects were instructed to release from plugging their nose and resume breathing. The expiratory pause was recorded with the stopwatch from the time the subject plugged their nose to the time at which they released or inhaled, whichever occurred first. Subjects were allowed to practice the expiratory pause 1–2 times before the pause was recorded. The expiratory pause was then recorded three times, and the best of these recordings was used for data analysis.

### 2.5. Data Collection and Analysis

Data were collected prior to and following the six-week DSB program. Data were collected at the same time period for the control and experimental groups. WOMAC scores, HRV, and expiratory pauses were obtained and recorded. Posttest data were collected 48 hours after the end of the DSB program.

Analysis was separated into three facets: subjective assessment using WOMAC scores (pain and physical), objective assessment using HRV variables (LF, HF, and LF to HF ratio), and evaluation of the training program using the expiratory pause. Change scores were calculated for each variable by subtracting the preintervention measurement from the postintervention measurement. Variables were analysed using three separate mixed ANOVAs. Change scores were analysed using a one-way ANOVA. The alpha level for analysis was set at less than 0.05. Effect sizes were also calculated. Statistical analysis was used to evaluate if there was an interaction between group and time or a main effect for group or time. Data analysis was completed with Excel and IBM SPSS 25 [25].

## 3. Results

### 3.1. Subject Characteristics

Twenty subjects (14 female, 6 male), aged 20–82 (67 ± 9) years old, participated in this study. Subjects had been diagnosed with osteoarthritis of their hip or knee (*n* = 13) or received a normative score of less than 50 on the American Academy of Orthopaedic Surgeons (AAOS) Hip and Knee Questionnaire (*n* = 7). The two younger subjects in this study had been diagnosed with osteoarthritis secondary to sports-related injuries. Subject characteristics are presented in Table 1.

### 3.2. Subjective Outcomes: WOMAC VAS Pain and Physical Function

For WOMAC pain scores, there was not a significant interaction between group and time (*p*=0.191, *η*_*p*_^2^ = 0.093) and there was not a significant main effect for group (*p*=0.812, *η*_*p*_^2^ = 0.003). There was a significant main effect for time (*p*=0.001), and the effect size was large (*η*_*p*_^2^ = 0.454). For WOMAC physical function scores, there was not a significant interaction between group and time (*p*=0.848, *η*_*p*_^2^ = 0.002) and there was not a significant main effect for group (*p*=0.671, *η*_*p*_^2^ = 0.01). There was a significant main effect for time (*p* < 0.001), and the effect size was large (*η*_*p*_^2^ = 0.506). Means and standard deviations for pain and physical function are presented in Table 2.

**Figure 1:**
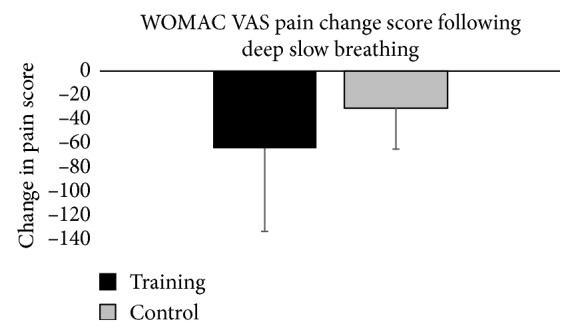
Graphical representation of change in WOMAC pain scores from pretest to posttest for each group. The error bars represent the standard deviation of measurements.

**Figure 2:**
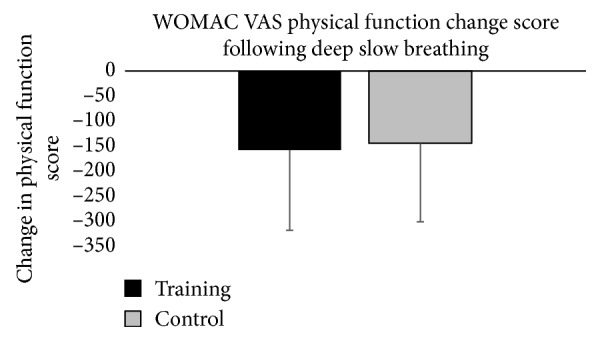
Graphical representation of change in WOMAC physical function scores from pretest to posttest for each group. The error bars represent the standard deviation of measurements.

Changes scores were not statistically significant for pain (*p*=0.191, *η*_*p*_^2^ = 0.093) or physical function (*p*=0.848, *η*_*p*_^2^ = 0.002). Means and standard deviations for pain and physical function change scores are presented in Figures 1 and 2. The change scores indicated that pain and physical function scores decreased for both training and control groups, but the changes were not statistically significant. Higher WOMAC scores indicate worse pain and physical function. The decreases in pain and physical function scores signify decreases in pain and improvements in physical function.

### 3.3. Objective Outcomes: LF Power, HF Power, and LF/HF Ratio

For LF power, there was not a significant interaction between group and time (*p*=0.478, *η*_*p*_^2^ = 0.03). There was not a significant main effect for group (*p*=0.418, *η*_*p*_^2^ = 0.039) or for time (*p*=0.306, *η*_*p*_^2^ = 0.061). For HF power, there was not a significant interaction between group and time (*p*=0.945, *η*_*p*_^2^ < 0.001). There was not a significant main effect for group (*p*=0.419, *η*_*p*_^2^ = 0.039) or for time (*p*=0.417, *η*_*p*_^2^ = 0.039). For LF/HF ratio, there was not a significant interaction between group and time (*p*=0.439, *η*_*p*_^2^ = 0.036). There was not a significant main effect for group (*p*=0.439, *η*_*p*_^2^ = 0.036) or for time (*p*=0.08). Means and standard deviations for LF power, HF power, and LF/HF ratio are presented in Table 3.

**Figure 3:**
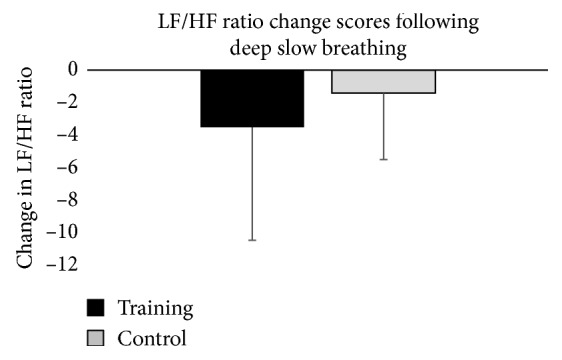
Graphical representation of change in LF/HF ratio from pretest to posttest for each group. The error bars represent the standard deviation of measurements.

Change scores for HRV indicate that there were no significant differences between the training and control groups. Change scores were not statistically significant for LF power (*p*=0.478, *η*_*p*_^2^ = 0.030), HF power (*p*=0.945, *η*_*p*_^2^ <0.001), or LF/HF ratio (*p*=0.439, *η*_*p*_^2^ = 0.036). Means and standard deviations for HRV change scores are presented in Table 3 and Figure 3.

### 3.4. Training Outcomes: Expiratory Pause

Expiratory pause values were similar for training and control groups. There was not a significant interaction between group and time (*p*=0.586, *η*_*p*_^2^ = 0.017). There was not a significant main effect for group (*p*=0.418, *η*_*p*_^2^ = 0.037), and there was not a significant main effect for time (*p*=0.743, *η*_*p*_^2^ = 0.006). Means and standard deviations for the expiratory pause are presented in Table 4.

**Figure 4:**
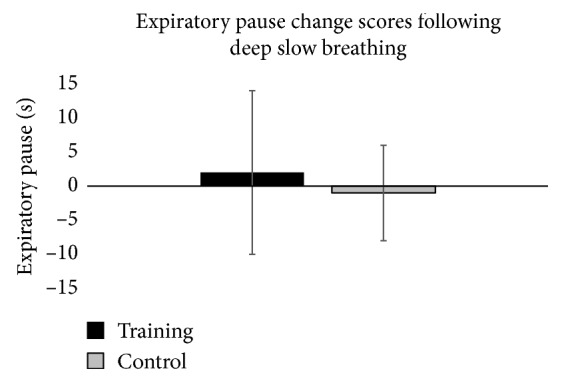
Graphical representation of change in expiratory pause from pretest to posttest for each group. The error bars represent the standard deviation of measurements.

Change scores were not statistically significant for the expiratory pause (*p*=0.569, *η*_*p*_^2^ = 0.018). Change scores for the expiratory pause are displayed in Figure 4.

### 3.5. Subject Compliance

Nine of the ten training subjects completed all weekly breathing logs. The mean amount of time spent practicing DSB at home each week was 118 minutes with a standard deviation of 22 minutes. All training group subjects attended each weekly training session at the senior center, and no training group subjects changed their medications during the study.

## 4. Discussion

The purpose of this study was to evaluate the effects of a six-week DSB exercise program on pain, physical function, and HRV in subjects with lower extremity joint pain. The goal of the DSB exercise program was to lengthen the exhalation phase of breathing and the pause following exhalation.

### 4.1. Subjective Outcomes: WOMAC VAS Pain and Physical Function

In our study, the DSB training program did not significantly alter pain and physical function in the DSB group compared with the control group. However, both training and control groups showed significant changes in WOMAC pain and physical function scores from pretest to posttest assessments. Additionally, the mean changes in pain and physical function met the MCII criteria [22]. However, the standard deviations for pain and physical function scores in both groups were very large and make it difficult to draw conclusions with regards to MCII criteria. Results indicate that while there were significant changes in pain and physical function scores, these changes did not result from the DSB exercise program. Alternatively, the changes in pain and physical function from pretest to posttest may be due to another variable affecting both training and control groups.

It is possible that subjects benefited from the social support they received while participating in the study. Social support may play an important role in improving pain and physical function for those suffering with OA [26–30]. In studies evaluating the relationship between social support and OA outcomes, social support protected subjects against poor WOMAC outcomes [30] and was associated with higher physical functioning, general health, mental health, social functioning, and vitality [27]. In addition to social support, optimism may help to mediate pain. Ferreira and Sherman [29] investigated the role of social support in well being and found that social support partially mediated pain perceptions in relation to depressive symptoms while optimism partially mediated pain perceptions in relation to life satisfaction. Both social support and optimism seem to be important mediators of OA pain [28].

The importance of social support may offer an explanation for the significant decrease in pain and improvement in physical function seen in both groups in the present study. Results of the present study combined with previous research highlight the potential importance of psychosocial factors in the adjustment to chronic pain. These results support a biopsychosocial model of pain. While the DSB exercise program was not sufficient to significantly alter WOMAC pain and physical function scores between groups, social support may have played a role in the improvements seen in both training and control groups. There is currently no research found that evaluates the use of research as a dimension of social support. More research is needed to confirm the theory that participation in a research study may increase perceived social support and improve self-reported OA outcomes.

### 4.2. Objective Outcomes: LF Power, HF Power, and LF/HF Ratio

In this study, the DSB training program did not significantly alter ANS activity in the training group compared with the control group. Results of the present study contrast with previous studies which indicate that DSB decreased S-ANS activity [8, 31, 32] and increased P-ANS activity [9, 31, 32]. However, a caveat is made that LF as an indicator of sympathetic tone has been challenged, noting that parasympathetic influence may impact LF especially during slow breathing rates [33–35]. Due to the strong relationship between pain and ANS activity [8, 9], it is surprising that the significant changes in WOMAC pain scores in the present study were not accompanied by significant changes in ANS activity. However, pain was self-reported in the present study. Studies supporting the relationship between pain and ANS activity have objectively measured pain perception by measuring heat pain threshold and tolerance [8, 9]. Further research is needed to examine the relationship between self-reported pain and ANS activity.

In contrast to the present study, a recent study [36] demonstrated a significant (*p* < 0.05) impact of postexhalation pause on HF. However, this study collected HRV data during the breathing training cycles and did not have the latency of 48 hours after a longer breathing program of 6 weeks, as in the present study.

Discrepancies between the present study and other research may be due to the methodology. This study measured HRV within 48 hours of the last at-home breathing exercise session. Subjects were allowed to breathe naturally during HRV recording. Many studies evaluating the effect of breathing on HRV use controlled breathing during HRV recording or measured HRV immediately after the DSB session [8, 9]. Chandla et al. [31] studied the effects of a six-week DSB program, using pranayama, and found significant increases in HF power and decreases in LF power and LF/HF ratio. However, they did not report the procedures used during HRV recording, and they did not identify the timing between last training and data collection. It is unknown if subjects breathed naturally or used controlled breathing during HRV recording for this study.

Breathing patterns during HRV recording may significantly impact HRV results. In a study of 24 healthy males, the effect of different breathing patterns on HRV was assessed. Results indicated that the breathing pattern had a moderate effect on LF power (*η*_*p*_^2^ = 0.125) and LF/HF ratio (*η*_*p*_^2^ = 0.082) and a strong effect on HF power (*η*_*p*_^2^ = 0.204) [32]. These results are supported by a study which suggests that changes in HRV at variable respiratory frequencies (0.15–0.5 Hz) may be due to respiration. Beda et al. suggest that respiratory parameters such as respiratory period, tidal volume, and the tidal volume power account for 79 percent of HRV changes [37]. These results reveal the relationship between respiratory variability and HRV and suggest that it is important to measure and assess respiration with HRV.

The methods of Kulur et al. may be an effective way of measuring the effects of a DSB program on HRV. Kulur et al. trained subjects to breathe at a rate of six respiratory cycles per minute, five seconds for inhalation and five seconds for exhalation, during HRV recording. During the intervention, subjects were trained with diaphragmatic breathing and follow-up recording was completed at three months and one year [38]. This methodology may help control for the changes in autonomic activity associated with respiratory variability during HRV recording.

Due to the influence of respiration on HRV [38–40], controlling for respiratory variability during preintervention and postintervention HRV data collection may provide a more accurate assessment of the effects of a breathing intervention on HRV. Previous research on the relationship of respiratory variability and HRV may partially explain the lack of significant changes in HRV in the present study. Furthermore, the large standard deviations seen in the training group may be the result of greater respiratory variability due to increased breathing awareness [40]. While breathing patterns may impact changes in HRV, it is important not to dismiss HRV results in studies without controlled breathing. Although the present study did not show significant changes in LF/HF ratio, there was a moderate effect size for time (*η*_*p*_^2^ = 0.169) for LF/HF ratio. The moderate effect size suggests that both training and control groups shifted toward more parasympathetic activity during the course of the study. More research is needed to examine the effects of DSB programs on HRV, utilizing controlled breathing during HRV data collection.

### 4.3. Training Outcome: Expiratory Pause and Anecdotal Outcomes

Results from this study demonstrate that the DSB exercise did not effectively increase the expiratory pause. While there is anecdotal evidence that the expiratory pause may be increased through training [12], there is no scientific evidence supporting this claim. This may explain why there were no significant changes in the expiratory pause during the study. More research is needed to determine if the expiratory pause can be increased and the amount of training and time needed to induce changes in the expiratory pause.

In addition to measured outcomes of this study, anecdotal results were also recorded. While no control subjects reported perceived changes during the study, eight training subjects reported a decrease in their stress level and an increase in their perceived ability to effectively manage stress. Eight subjects reported that the breathing exercises helped them to cope with their joint pain, even when the pain did not subside following the DSB exercises. Eight training subjects reported increased awareness of both their breathing patterns and awareness of tension in their bodies. All training subjects reported being able to effectively release tension in their body and relax using the breathing exercises.

### 4.4. Limitations

Limitations of this study involve the lack of control for other variables such as age, body mass index, knee pain intensity, and disease severity. The small, nonrandom nature of the sample may have also affected the results and limited the generalizability of the results. The small sample size may have resulted in large standard deviations of all variables in this study.

## 5. Conclusion

Six weeks of DSB did not significantly alter pain-related variables in subjects with lower extremity joint pain. However, both training and control groups experienced significant decreases in pain and significant improvements in physical function over the course of the study. Changes in pain and physical function appear to be the result of social support that subjects received by participating in the study. The lack of significant changes in HRV variables may have been due to the large standard deviations and methodology used during HRV data collection. Further research is needed as the present study is the first to evaluate the use of DSB as an intervention for arthritis-related pain and physical limitation. This research will provide more information about the role of DSB as an intervention for joint pain. A better understanding of the effects and mechanisms of DSB may provide novel and practical approaches to treating joint pain. Future research would ideally identify subjects with arthritis who would benefit most from DSB and specify the amount of DSB needed to produce clinically important improvements in pain and physical function. At this time, DSB does not appear to produce significant changes in OA joint pain or physical function.

## Figures and Tables

**Table 1:** Subject characteristics (mean ± standard deviation (SD)).

Subject characteristics	Training (*n* = 10)	Control (*n* = 10)
Age (years)	67 ± 9	48 ± 19
Height (m)	1.6 ± 0.1	1.7 ± 0.1
Weight (kg)	70.5 ± 13.6	73.8 ± 13.4

**Table 2:** WOMAC pain and physical function scores (mean ± SD) and changes in WOMAC pain and physical function scores (mean ± SD) for each group before and after the test.

	Pretest	Posttest
Pain		
** **Training	148 ± 94	84 ± 89
** **Control	123 ± 87	92 ± 73

Physical function		
** **Training	434 ± 268	276 ± 287
** **Control	482 ± 346	338 ± 274

Change in WOMAC scores	Pain	Physical function
** **Training	−64 ± 69	−158 ± 160
** **Control	−31 ± 34	−144 ± 155

**Table 3:** Heart rate variability: low frequency power, high frequency power, and sympathetic/vagal ratio (mean ± SD) for each group before and after the test and changes in LF power, HF power, and LF/HF ratios (mean ± SD) for each group.

	Pretest	Posttest	
Low frequency power (ms^2^)			
** **Training	179.30 ± 562.1	9.82 ± 28.84	
** **Control	32.08 ± 66.55	0.74 ± 0.73	

High frequency power (ms^2^)			
** **Training	14.04 ± 39.76	7.02 ± 19.24	
** **Control	7.43 ± 9.99	1.50 ± 1.87	

Sympathetic/vagal ratio			
** **Training	5.64 ± 10.03	2.13 ± 3.72	
** **Control	2.38 ± 3.79	0.97 ± 0.69	

Changes in HRV scores	LF power (ms^2^)	HF power (ms^2^)	LF/HF ratio
** **Training	−169.57 ± 566.52	−7.02 ± 45.78	−3.51 ± 6.93
** **Control	−31.34 ± 66.36	5.93 ± 8.95	−1.41 ± 4.07

**Table 4:** Expiratory pause (mean ± SD) for each group before and after the test and expiratory pause change score (mean ± SD) for each group.

	Pretest	Posttest
Expiratory pause (s)		
** **Training	21 ± 12	23 ± 9
** **Control	26 ± 10	25 ± 8

Expiratory pause change score (s)		
** **Training	2 ± 12	
** **Control	−1 ± 7	

## Data Availability

The data used to support the findings of this study are available from the corresponding author upon request.

## Supplementary Materials

Supplementary Materials Video demonstrations of the full and modified expiratory pauses that were provided to the study participants as reminders for them to review.
